# Utilization of Dental Care, Tooth Loss, and Oral Health-Related Quality of Life in Older Adults Visiting Dental Care Centers in Indian Settings

**DOI:** 10.7759/cureus.31128

**Published:** 2022-11-05

**Authors:** Kamal Nayan, Abdul Azam Khan, Pallavi Kusum, Prakash Kumar, Lalima Kumari, Srijan K Srivastav

**Affiliations:** 1 Department of Prosthodontics, Mithila Minority Dental College and Hospital, Darbhanga, IND; 2 Department of Oral and Maxillofacial Surgery, Dental College Azamgarh, Azamgarh, IND; 3 Department of Orthodontics and Dentofacial Orthopaedics, Budhha Institute of Dental Sciences & Hospital, Patna, IND; 4 Department of Conservative Dentistry and Endodontics, Mithila Minority Dental College and Hospital, Darbhanga, IND; 5 Department of Orthodontics and Dentofacial Orthopaedics, Patna Dental College and Hospital, Patna, IND; 6 Department of Oral and Maxillofacial Surgery, Subharti Dental College & Hospital, Meerut, IND

**Keywords:** tooth loss, quality of life, periodontal disease, older adults, general oral health assessment index, dental caries

## Abstract

Background: Oral disease prevalence is rising globally in a major way, with a heavier impact and concern on developing countries like India. Also, limited data in literature has previously assessed oral health-related quality of life (OHRQoL) in older adults in Indian settings.

Aims: The present study aimed to evaluate OHRQoL in older adults seeking dental care in Indian settings.

Methods: In 140 subjects, an oral examination to detect any oral condition was done followed by filling out a questionnaire for OHRQoL assessment using the Geriatric Oral Health Assessment Index 12 (GOHAI-12). The treatment needs of each study subject were governed by prosthetic and dental status individually. Statistical evaluation of the data collected was done to formulate the results.

Results: Concerning age, a significant impact was seen, where increasing age was associated with poor quality of life (p=0.025). For gender, older females had poor OHRQoL with p-values of <0.001, 0.01, 0.04, and <0.001 respectively for behaviour, psychological, pain and discomfort, and functional limitation. Also, edentulous patients had poorer OHRQoL compared to subjects with >20 teeth

Conclusion: The present study concludes that oral diseases can significantly affect the OHRQoL, with higher female and edentulous state preponderance. Early diagnosis and management can aid in improving QoL in older adults.

## Introduction

Health is the main concern for healthcare professionals, and it is always taken as the basic right of human beings. Health is also a broad social goal globally, including in India. Oral health is also a vital component of the oral well-being and health of both adults and children. However, dental health is sometimes disregarded as an important aspect of overall health. Following the WHO guidelines and statement, oral health goes beyond just healthy dentition. A healthy oral cavity, according to WHO, is free of oral cavity disorders and diseases such as tooth loss, tooth decay, periodontal diseases, cleft lip and cleft palate birth defects, oral sores, throat and oral cancer, and/or chronic facial and mouth pain [1].

Diseases affecting dentition are usually not self-limiting entities. When not treated, dental situations can affect the overall quality of life and the overall well-being of the affected subjects. Regular health checkups and daily home oral care are vital steps to preserving the health of the teeth and overall dentition. Dental services can be defined as the first reported dental visit within a visit series or dental visits lacking in a specific time frame or annual dental visits per individual or per person proportionally visiting a dentist in a year. In a few countries, dental services are also assessed by average expenses for routine or emergency dental visits. Assessing these services also helps in implementing and planning oral health services at the community level [1,2].

In developing countries like India, the commonly reported oral health care concerns, including oral cancers, periodontitis, and dental caries, pose a high concern among the community. They also affect the social lives of the affected subjects by imposing restrictions on their home, work, and school lives, resulting in a reduced quality of life in subjects of all age groups. The quality of life is more severely impacted in subjects with low socioeconomic status. In developing countries like India, the difference in oral health is significantly high in the rural and urban populations. Although various advances have been reported in dental technologies, procedures, and materials, there is limited access to oral health care for the sub-groups of the population [2,3]. Oral health is poorer in older adults owing to the cumulative and chronic nature of various conditions affecting oral status. These include tooth loss, periodontal diseases, and dental caries, as these are commonly encountered oral diseases. Various conditions concerning general health such as diabetes and cardiovascular diseases can also lead to increased oral diseases in an individual, including periodontal disease, altered taste, xerostomia, and others. Tooth loss, common in older individuals, can lead to compromised esthetics, phonetics, and chewing difficulties. Difficulty in chewing can lead to compromised nutrition uptake, which can proceed to a debilitating stage if prolonged [1].

The older population comprises a large portion of the overall population globally, owing to increased life expectancy and improved healthcare structures worldwide. The elderly population is expected to increase by two billion by the end of 2050. In India, older adults account for 9% (103 billion) of the total population. The prevalence of caries in India is 31 to 100% [2]. Promoting oral health in India is focused on diagnosing prevalent oral conditions in the targeted population. However, emphasis on special needs and characteristics is also vital. Quality of life is not accurately judged by disease prevalence. Hence, oral health assessment should include oral health-related quality of life (OHRQoL), which is considered a vital part of the global oral health programme by WHO [3]. OHRQoL can be assessed and measured using various indices, with the Oral Health Impact Profile and General Oral Health Assessment Index (GOHAI) being the most reliable and commonly used. GOHAI is also available in Hindi, which makes it possible to use in India and makes it easier to use [4]. Also, limited data in the literature has previously assessed OHRQoL in older adults in the Indian scenario [5-7]. The present study aimed to evaluate OHRQoL in older adults seeking dental care in Indian settings.

## Materials and methods

The study was carried out after obtaining clearance from Mithila Minority Dental College, Darbhanga, India, and informed consent from the study participants. The study included a total of 140 subjects from both genders with a mean age of 71.46 years and an age range of 60-87 years. MMDC/2021/102 was the ethical number. The population sample for the present study were the patients from the Outpatient Department of Mithila Minority Dental College and Hospital, Darbhanga, India, who came for their dental care needs. The exclusion was done for subjects who were terminally ill, had morbidity, had systemic diseases, or were not willing to participate in the study. A total of 226 subjects were screened, and 140 subjects were finally included. After final inclusion, all the subjects underwent an oral examination to detect any oral condition, if present. This was followed by an interview concerning the impact of oral conditions on OHRQoL. All oral examinations and interviews were taken by a single examiner with expertise in the field. Oral/mucosal lesions, prosthetic status, and the Decayed, Missing, Filled (DMF) Index for caries were used for assessing oral needs, and the Community Periodontal Index (CPI) for periodontal needs assessment was used. Individual treatment needs were also assessed.

This was followed by filling out the questionnaire. The questions of the questionnaire were provided in both Hindi and English for better understanding. The questionnaire was comprised of questions regarding demographic characteristics, socioeconomic status, family history, educational history, personal habits, medical history, dental problems, oral health, and dental awareness. GOHAI-12 was used for assessing OHRQoL. The OHRQoL assessment using GOHAI-6 focused on 9 items and had 12 questions assessing talking, swallowing, and eating for G1, G2, G3, and G4. There are five responses to each question, including 0 = never, 1 = seldom, 2 = sometimes, 3 = often, 4 = very often, and 5 = always. G6, G7, G9, G10, and G11 assessed psychological aspects, including oral health worries, social withdrawal, and self-esteem. G5, G8, and G12 assessed the use of pain-relieving drugs and discomfort. The overall scores ranged from 0 to 60, where 60 was the maximum score showing good oral health. The influence of oral health status on health-related quality of life (HRQoL) was assessed based on the scores of the questionnaire. These scores evaluated the psychosocial and functional consequences of oral health on HRQoL.

The data records were assessed statistically with IBM SPSS Statistics for Windows, Version 22.0 (Released 2013; IBM Corp., Armonk, New York, United States) and analysis of variance (ANOVA) test. The level of significance was kept at a p-value of 0.05. The data were expressed as a mean, number, and percentage, and the outcomes were calculated.

## Results

The study included a total of 140 subjects from both genders with a mean age of 71.46 years and an age range of 60-87 years. The demographic data of the subjects from the present study is listed in Table 1. The study included 83.57% (n = 117) subjects from the age range of 60-70 years, 12.14% (n = 17) from 71-80 years, and 4.28% (n = 6) above 80 years. There were 75.71% (n = 106) females and 24.28% (n = 34) males. 41.42% (n = 58) of subjects were employed and 58.57% (n = 82) of subjects were unemployed. 29.28% (n = 41) were smokers and 70.71% (n = 99) were non-smokers. A BMI of >19 was seen in 62.14% (n = 87) of subjects. A total of 62.85% (n = 88) used a toothbrush with toothpaste for oral hygiene.

**Table 1:** Demographics of the study subjects

Characteristics	Subgroups	%	n
Mean age (years)		71.46±4.94	
Age range (years)	60-70	83.57	117
	71-80	12.14	17
	Above 80	4.28	6
Gender	Females	75.71	106
	Males	24.28	34
Occupational status	Employed	41.42	58
	Unemployed	58.57	82
Educational status	Primary/less	62.14	87
	Undergraduate	24.28	34
	Postgraduate	13.57	19
Smoking habit	Smokers	29.28	41
	Non-smokers	70.71	99
BMI	<19	37.85	53
	>19	62.14	87
Oral hygiene methods	Toothbrush with paste	62.85	88
	Finger	9.28	13
	Toothpowder	27.85	39
Tobacco chewing history	Present	68.57	96
	Not present	31.42	44

On assessing the oral health and treatment needs, it was seen that 65.71% (n = 92) of subjects had no caries and >2 carious lesions were found in 30% (n = 42) of the subjects. Leukoplakia, ulcerations, oral submucous fibrosis (OSMF), candidiasis, and lichen planus were seen in 3.57% (n = 5), 0.71% (n = 1), 42.14% (n = 59), 30% (n = 42), and 2.85% (n = 4) subjects, respectively. Clinical attachment loss (CAL) of >5mm was seen in 25% (n = 35) of the subjects. Buccal mucosa, lip, palate, tongue, and floor of mouth lesions were seen in 40% (n = 56), 3.57% (n = 5), 0.71% (n = 1), 30% (n = 42), and 2.84% (n = 4) of the subjects, respectively. Concerning treatment needs, emergency care was needed by 18.57% (n = 26) of subjects. Intervention was needed by 45.71% (n = 64) subjects, preventive care by 34.28% (n = 48) subjects, and 1.42% (n = 2) needed referral to a specialist (Table 2).

**Table 2:** Oral status and treatment needs in the study subjects

Condition	Parameters	%	N
Teeth present	Edentulous	42.85	60
	<20	40	56
	>20	17.14	24
Caries	None	65.71	92
	<2	4.28	6
	>2	30	42
Oral lesions	None	20.71	29
	Leukoplakia	3.57	5
	Ulcers	0.71	1
	OSMF	42.14	59
	Candidiasis	30	42
	Lichen planus	2.85	4
Clinical attachment loss (CAL)	None	29	41
	<5mm	46	64
	>5mm	25	35
Sites of lesions	Buccal mucosa	40	56
	Lip	3.57	5
	Palate	0.71	1
	Tongue	30	42
	Floor of mouth	2.84	4
	Others	22.85	32
Treatment required	Immediate intervention (abscess/pain)	18.57	26
	Intervention needed	45.71	64
	Only preventive measures	34.28	48
	Referred	1.42	2

The GOHAI assessment based on age, gender, and dentition was used to assess the OHRQoL. It was seen that, concerning age, there was a significant impact, where increasing age was associated with poor quality of life (p = 0.025). For gender, older females had poor OHRQoL with p-values of <0.001, 0.01, 0.04, and <0.001 respectively for behaviour, psychological, pain and discomfort, and functional limitation. Also, edentulous patients had poorer OHRQoL compared to subjects with >20 teeth (Table 3).

**Table 3:** GOHAI assessment based on age, gender, and dentition in study subjects

GOHAI component	n=117	n=17	n=6	P-value
Age-based	60-70 years	71-80 years	>80 years	
Behaviour impact	3.58±1.67	3.24±1.82	4.82±2.49	Not significant
Psychological impacts	6.72±2.24	6.89±2.34	8.9±1.68	Not significant
Pain and discomfort	5.25±1.85	5.11±1.95	4.9±2.37	0.025
Functional limitation	5.4±2.21	5.11±1.95	4.9±2.37	N.S
Gender-based	Females (n=106)	Males (n=34)		
Behaviour Impact	3.166±1.399	4.90±1.988		<0.001
Psychological impacts	6.48±2.408	7.75±2.789		0.01
Pain and discomfort	5.12±1.822	5.88±2.060		0.04
Functional limitation	4.84±1.962	7.34±1.681		<0.001
Dentition-based	Edentulous (n=60)	<20 (n=56)	>20 (n=24)	
Behaviour Impact	3.8±1.90	3.21±1.52	3.4±1.56	0.00
Psychological impacts	6.92±2.41	3.92±1.91	7.03±2.28	Not significant
Pain and discomfort	5.35±2.15	5.06±1.73	5.76±58	Not significant
Functional limitation	6.19±2.47	4.64±1.67	5.37±1.82	0.00

## Discussion

Dental services are utilised by a limited population in a limited manner, especially in the population visiting community healthcare centres, particularly in developing countries like India. Also, health insurance services in India are nonexistent. And when they exist, they are not utilised by the majority of the population as they reside in rural areas or areas with limited access to and awareness of these services [8]. In the present study, OHRQoL was utilised. OHRQoL assesses the comfort of the subjects during social interaction, engagement, sleeping, and eating. OHRQoL also assesses the satisfaction of the subjects with their oral health and related needs. Various advances have been made in the recording of OHRQoL and related tools and scales [9,10].

One such scale is GOHAI, which assesses the patient-centred definition of health, which diverges from disease-centred epidemiological measures of health. GOHAI was initially developed in 1990 by Atchinson and Dolan and was utilised for the geriatric population of North America. It is considered a reliable tool as it is widely used, stable, and available in many languages, including Hindi, which was advantageous for Indian subjects' use. Atchinson in 1996 also reported that the Oral Health Assessment Index was assessed for various races, ages, genders, and communities and was found to be reliable following its renaming as GOHAI [11,12]. The present study assessed 140 elderly subjects with a mean age of 71.46 years. The majority of the study subjects were in the age range of 60-70 years with 83.57% (n =117) subjects and the least in >80 years with 4.28% (n =6) subjects. In the present study, more females were the study subjects compared to males. The majority of the study subjects were non-smokers and practised good oral hygiene. These demographic data were the same as what was found by Chahar et al. [13] in 2019 and Bhat et al. [14] in 2010 when they looked at similar data.

For evaluation of treatment needs and oral health, no caries was seen in the highest proportion of study subjects with 65.71% (n = 92) subjects, and in 30% (n = 42) subjects, more than 2 carious lesions were seen. Lichen planus, candidiasis, OSMF, ulcerations, and leukoplakia were seen in 2.85% (n = 4), 30% (n = 42), 42.14% (n = 59), 0.71% (n = 1), and 3.57% (n = 5) subjects, respectively. Involvement of the floor of the mouth, tongue, palate, lip, and buccal mucosa was seen in the study subjects. For the treatment required, 1.42% (n = 2) needed referral to a specialist, 34.28% (n = 48) subjects needed preventive care, and 45.71% (n = 64) subjects needed intervention. These results were consistent with the results of Bhatt et al. [15] in 2011 and Kumar et al. [16] in 2009, where authors reported similar treatment needs and oral health status.

OHRQoL was assessed using dentition, gender, and age, and it was seen that poor quality of life was associated with increasing age (p = 0.025). Functional limitation, pain and discomfort, psychological, and behavioural p-values were 0.001, 0.04, 0.01, and 0.001, respectively for poor OHRQoL in older females. Poorer OHRQoL was seen in edentulous subjects compared to dentate subjects. These findings were similar and comparable to the results of Lolita et al. [17] in 2015 and Joseph et al. [18] in 2016, as authors in their respective studies described female preponderance and the edentulous state as a reason for poor OHRQoL.

## Conclusions

Within its limitations, the present study concludes that oral diseases can significantly affect OHRQoL, with a higher female and edentulous state preponderance. Early diagnosis and management can aid in improving QoL in older adults, thereby promoting better general health. Specific programmes should be made to screen and evaluate oral health at the early stages of life in geriatric subjects. However, the limitations of the present study were cross-sectional design, a smaller sample size, and a smaller follow-up interval. Hence, more prospective studies are warranted to reach a decisive conclusion.
